# Association of hypertension and incident diabetes in Chinese adults: a retrospective cohort study using propensity-score matching

**DOI:** 10.1186/s12902-021-00747-0

**Published:** 2021-04-29

**Authors:** Yang Wu, Haofei Hu, Jinlin Cai, Runtian Chen, Xin Zuo, Heng Cheng, Dewen Yan

**Affiliations:** 1 Department of Endocrinology, The First Affiliated Hospital of Shenzhen University, No.3002 Sungang Road, Futian District, Shenzhen, 518035 Guangdong Province China; 2 Department of Endocrinology, Shenzhen Second People’s Hospital, Shenzhen, 518035 Guangdong Province China; 3 Shenzhen University Health Science Center, Shenzhen, 518071 Guangdong Province China; 4 Department of Nephrology, The First Affiliated Hospital of Shenzhen University, Shenzhen, 518035 Guangdong Province China; 5 Department of Nephrology, Shenzhen Second People’s Hospital, Shenzhen, 518035 Guangdong Province China; 6 Shantou University Medical College, Shantou, 515000 Guangdong Province China; 7 Department of Endocrinology, The Third People’s Hospital of Shenzhen, Shenzhen, 518116 Guangdong Province China

**Keywords:** Hypertension, Incident diabetes, Propensity-score matching, Inverse probability of treatment weights, Risk

## Abstract

**Background:**

Reliable quantification of the relationship between hypertension and diabetes risk is limited, especially among Chinese people. We aimed to investigate the association between hypertension and the risk of diabetes in a large cohort of the Chinese population.

**Methods:**

This was a retrospective propensity score-matched cohort study among 211,809 Chinese adults without diabetes at baseline between 2010 and 2016. The target independent and dependent variable were hypertension at baseline and incident diabetes during follow-up respectively. The propensity score matching using a non-parsimonious multivariable logistic regression was conducted to balance the confounders between 28,711 hypertensive patients and 28,711 non-hypertensive participants. The doubly robust estimation method was used to investigate the association between hypertension and diabetes.

**Results:**

In the propensity-score matching cohort, diabetes risk increased by 11.0% among hypertensive patients (HR = 1.110, 95% confidence interval (CI): 1.031–1.195, *P* = 0.00539). And diabetes risk dropped to 8.3% among hypertensive subjects after adjusting for the propensity score (HR = 1.083, 95%CI: 1.006–1.166, *P* = 0.03367). Compared to non-hypertensive participants with low propensity score, the risk of incident diabetes increased by 2.646 times among hypertensive patients with high propensity score (HR = 3.646, 95%CI: 2.635–5.045, *P* < 0.0001).

**Conclusion:**

Hypertension was associated with an 11.0% increase in the risk of developing diabetes in Chinese adults. And the figure dropped to 8.3% after adjusting the propensity score. Additionally, compared to non-hypertensive participants with low propensity scores, the risk of incident diabetes increased by 2.646 times among hypertensive patients with high propensity scores.

**Supplementary Information:**

The online version contains supplementary material available at 10.1186/s12902-021-00747-0.

## Background

Diabetes mellitus is an important global public health problem with high morbidity and disability. The World Health Organization estimated that the prevalence of diabetes in adults was 8.5% in 2016 [1]. Due to the aging population and unhealthy lifestyles, the prevalence of diabetes worldwide tends to continue to rise. The global diabetes prevalence in 2019 was estimated to be 9.3% (463 million people), rising to 10.2% (578 million) by 2030 and 10.9% (700 million) by 2045 [2]. It is a debilitating chronic epidemic with potentially various complications. Diabetes can mediate multiple organ damage, leading to cardiovascular events, kidney disease and cerebrovascular complications [3–5]. Consequently, the high morbidity of diabetes has important social, economic and developmental implications worldwide.

American Diabetes Association (ADA) position statement showed that hypertension and diabetes common co-exist in the same individual, which depends on the type of diabetes, age, gender, race/ethnicity, body mass index, and the presence of kidney disease, among other factors [6–9]. The two diseases have etiological aspects in common, such as obesity, inflammation, oxidative stress, insulin resistance, and factors associated with increased microvascular and macrovascular impairment [10]. Diabetes mellitus is more frequent in hypertensive than normotensive subjects [11–13]. Therefore, hypertension may be considered among the greatest provoking factors for incident diabetes. Furthermore, uncontrolled blood pressure was associated with a two-fold increased risk of incident diabetes in treated hypertensive patients [14]. A study extending the findings showed the presence of hypertensive target organ damage increased the risk of developing diabetes [15]. Despite the evidence linking hypertension and incident diabetes, published studies on the impact of hypertension on the development of diabetes have provided conflicting findings. Although some studies have demonstrated an increased risk of diabetes in patients with hypertension, others have observed that after adjustment for some covariates, blood pressure has no significant effect on the risk of the subsequent development of diabetes [16–19]. Given these discrepant findings, most of these studies recruited a relatively small number of patients from a single center, and they did not ensure balance in measured confounders.

The traditional parsimonious regression model used in previous studies could result in bias because of unmeasured or residual confounding or the overfitting of the model [20], potentially preventing identification of the association between hypertension and incident diabetes. However, the propensity score is a conditional probability of having a particular exposure given a set of measured covariates at baseline. Propensity score matching is useful in such studies in which there are many covariates potentially confounding a rare outcome, and there are resource constraints that prevent the conduction of randomized clinical trials [21]. Therefore, a large cohort study, using propensity score-matched (PSM) data to estimate the association between hypertension and incident diabetes should be conducted, using real-world data from 211,809 Chinese adults across 32 sites and 11 cities between 2010 and 2016.

## Methods

### Study design and data source

This retrospective cohort study was based on a computerized database established by the Rich Healthcare Group in China, namely, the ‘DATADRYAD’ database (www.Datadryad.org). We downloaded the raw data for free from the site, provided by Chen et al. [22] from: Association of body mass index and age with incident diabetes in Chinese adults: a population-based cohort study. Dryad Digital Repository. 10.1136/bmjopen-2018-021768). The original study enrolled a total of 685,277 Chinese persons ≥20 years old with at least two visits from 2010 to 2016 across 32 sites and 11 cities in China (Shanghai, Beijing, Nanjing, Suzhou, Shenzhen, Changzhou, Chengdu, Guangzhou, Hefei, Wuhan, Nantong). Cohort entry was defined as the date of the initial visit. In each visit to the health check center, participants completed a detailed questionnaire assessing demographic, lifestyle and family history of chronic disease. The trained staff conducted the clinical measurements, including body weight, height, blood pressure. Biochemical tests about fasting plasma glucose (FPG), triglyceride (TG), total cholesterol (TC), high-density lipoprotein cholesterol (HDL-C), low-density lipoprotein cholesterol (LDL-C), serum creatinine (Scr), serum urea nitrogen (BUN), alanine aminotransferase (ALT) and aspartate aminotransferase (AST) were performed on an autoanalyzer (Beckman 5800). Body mass index (BMI) was equal to the weight divided by the square of height. The estimated glomerular filtration rate (eGFR) was calculated using the Chronic Kidney Disease Epidemiology Collaboration equation (CKD-EPI). The data were collected under standardized conditions and performed in accordance with uniform procedures. Laboratory methods also were carefully standardized through stringent internal and external quality controls.

The authors of the original study have waived all copyright and related ownership of the raw data. Therefore, we could use these data for secondary analysis without infringing on the authors’ rights. Furthermore, the original study was approved by the Rich Healthcare Group Review Board, and the information was retrieved retrospectively. And the original study was conducted in accordance with the Declaration of Helsinki, so did this secondary research [22]. The data are anonymous, and the requirement for informed consent was waived by the Rich Healthcare Group Review Board due to the observational nature of the study, as reported elsewhere [23, 24].

### Study sample

**Fig. 1:**
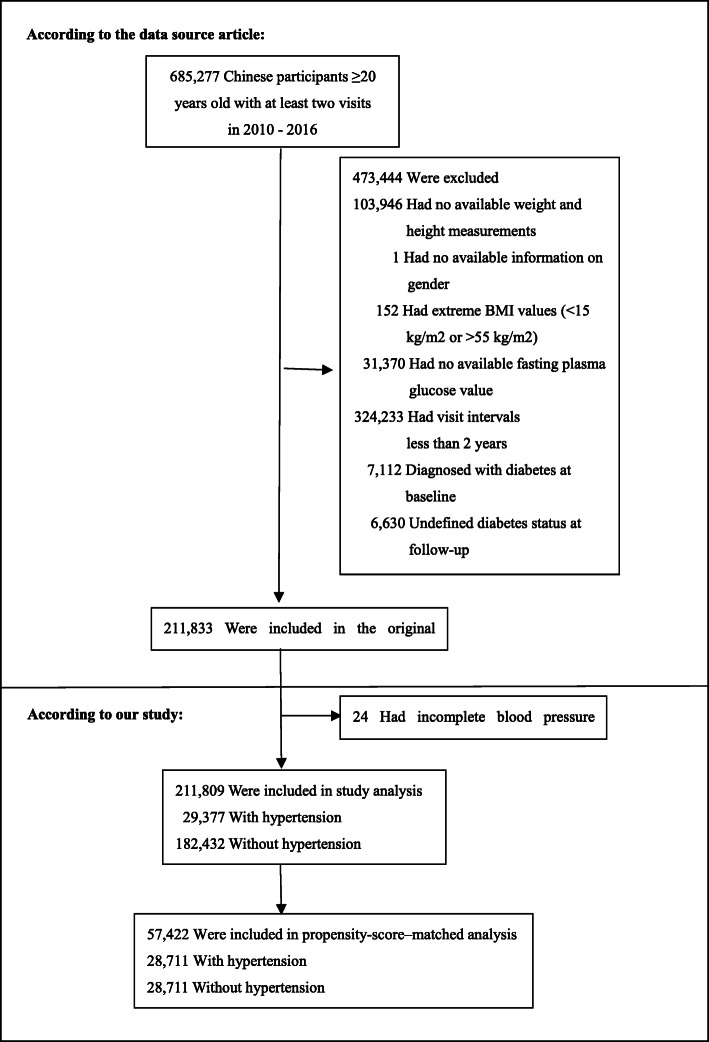
Flowchart of study participants

Consistent with the original study, participants were eligible for inclusion in our research aged 20–99 years old with at least two visits between 2010 and 2016. Participants were excluded at baseline in the original study, as follows:(1) no available information on weight, height and gender; (2) extreme BMI values (< 15 kg/m2 or > 55 kg/m2, 3) visit intervals < 2 years; (4) no available fasting plasma glucose value; (5) participants diagnosed with diabetes at baseline and participants with undefined diabetes status at follow-up. A total of 211,833 participants remained after applying the exclusion criteria in the original study [22]. In the present study, we further excluded participants with incomplete blood pressure (*n* = 24). Figure 1 depicted the participant’s selection process. Finally, our study included 211,809 participants for the secondary analysis. And the baseline characteristics of the included population and the excluded population were similar.

### Outcome measures

The outcome of interest was incident diabetes. Diabetes mellitus was defined as fasting plasma glucose ≥7.00 mmol/L and/or self-reported diabetes during the follow-up period [22]. Patients were censored at the time of diagnosis of diabetes or the last visit, whichever came first. Fasting venous blood samples were collected after at least 10 h fast at each visit. Plasma glucose levels were measured by the glucose oxidase method.

### Exposure of interest and covariates

The exposure of interest was hypertension. Hypertension was defined as systolic blood pressure (SBP) values ≥140 mmHg and/or diastolic blood pressure (DBP) values ≥90 mmHg [25–27]. Blood pressure value was obtained by trained staff using standard mercury sphygmomanometers through office blood pressure measurements. Covariates of interest included age, gender, BMI, FPG, TG, TC, HDL-C, LDL-C, ALT, AST, BUN, eGFR, smoking status, drinking status, family history of diabetes.

### Statistical analyses

Continuous variables were expressed as the means ± standard deviations (normal distribution) or medians (quartiles) (skewed distribution), and categorical variables were expressed as frequency or percentages. Two-sample t-tests were used for normally distributed continuous variables, Wilcoxon rank-sum tests for non-normally distributed continuous variables, and chi-square tests for categorical variables [28]. Missing continuous variables were mainly supplemented by means or median. And missing categorical variables in each covariate are considered as a group.

Considering the differences in the baseline characteristics between eligible participants in hypertension and non-hypertension groups (Table 1), propensity-score (PS) matching was used to identify a cohort of patients with similar baseline characteristics. The propensity score was estimated using a non-parsimonious multivariable logistic-regression model [29], with hypertension as the independent variable and all the baseline characteristics outlined in Table 1 as covariates. The variables used for matching included age, gender, BMI, FPG, TC, TG, HDL-C, LDL-C, ALT, AST, BUN, eGFR, family history of diabetes, smoking and drinking status. Matching was performed with the use of a 1:1 matching protocol without replacement (greedy-matching algorithm), with a caliper width equal to 0.0005. More stringent caliper was also attempted but 0.0005 gave the best matching model. Standardized differences (SD) were estimated for all the baseline covariates before and after matching to assess pre-matched imbalance and post-matched balance [30]. Standardized differences of less than 20.0% for a given covariate indicated a relatively small imbalance. The person-years of follow-up were calculated from the baseline interview to the date of incident diabetes or follow-up interview, whichever came first [31]. We used cumulative incidence and person-years incidence to describe the incidence rate [32]. Besides, we also used the log-rank test to compare the Kaplan–Meier hazard ratios (HR) for incident diabetes. The doubly robust estimation method, the combination of the multivariate regression model and a propensity score model, was also applied to infer the independent associations between blood pressure status and the risk of diabetes [33, 34]. The Cox proportional-hazards regression model was performed by adjusting for all covariates in the PS matched cohort. Prespecified subgroup analyses were performed on the basis of two types of characteristics. Subgroups were based on age, gender, BMI, FPG, eGFR, BUN, ALT, AST, TC, TG, HDL-C, LDL-C, smoking and drinking status. For the continuous variables, we converted them to a categorical variable according to the clinical cut point. Each stratification was adjusted for all the factors, except for the stratification factor itself. In the subgroup analyses, only the corresponding matched pairs in the same subgroup were chosen to maintain the balance of baseline characteristics between hypertension and non-hypertension groups. For example, in the subgroup of participants with FPG < 6.1 mmol/L, only when matched pairs of hypertensive and non-hypertensive participants both belong to the FPG < 6.1 mmol/L subgroup, these participants can be included in the subgroup analysis. The modifications and interactions of subgroups were inspected by likelihood ratio tests.

**Table 1:** Baseline characteristics before and after propensity-score matching in the original cohort

Characteristic		Before Matching			After Matching	
	Hypertension	Non-hypertension	SD (100%)	Hypertension	Non-hypertension	SD (100%)
Participants	29,377	182,432		28,711	28,711	
Age (years)	51.53 ± 14.84	40.58 ± 11.56	82.0	51.05 ± 14.57	48.60 ± 13.81	17.2
Gender			35.0			87.0
Male	20,410 (69.48%)	95,702 (52.46%)		19,858 (69.17%)	28,290 (98.53%)	
Female	8967 (30.52%)	86,730 (47.54%)		8853 (30.83%)	421 (1.47%)	
BMI (Kg/m2)	25.29 ± 3.41	22.91 ± 3.21	72.0	25.20 ± 3.35	25.19 ± 3.33	0.3
FPG (mmol/L)	5.15 ± 0.66	4.88 ± 0.59	43.0	5.14 ± 0.66	5.13 ± 0.64	1.6
TC (mmol/L)	5.00 ± 0.93	4.66 ± 0.87	37.0	4.99 ± 0.93	4.88 ± 0.93	12.0
TG (mmol/L)	1.43 (1.00–2.11)	1.03 (0.71–1.50)	44.0	1.420 (1.00–2.10)	1.43 (1.00–2.16)	4.2
ALT (U/L)	22.30 (16.00–34.00)	17.50 (12.50–26.10)	27.0	22.30 (16.00–34.00)	24.60 (17.90–37.00)	12.4
BUN (mmol/L)	4.90 ± 1.20	4.62 ± 1.11	24.0	4.89 ± 1.19	4.82 ± 1.11	5.4
eGFR (ml/min/1.73 m^2)	101.79 ± 17.04	111.38 ± 14.86	60.0	102.19 ± 16.85	111.83 ± 15.99	58.7
HDL-C (mmol/L)			12.0			18.0
<1.04	2539 (8.64%)	11,777 (6.46%)		2481 (8.64%)	3078 (10.72%)	
≥1.04	15,014 (51.11%)	87,923 (48.19%)		14,662 (51.07%)	12,104 (42.16%)	
Not recorded	11,824 (40.25%)	82,732 (45.35%)		11,568 (40.29%)	13,529 (47.12%)	
LDL-C (mmol/L)			14.0			13.7
<4.14	16,977 (57.79%)	97,295 (53.33%)		16,557 (57.67%)	14,838 (51.68%)	
≥4.14	938 (3.19%)	3184 (1.75%)		912 (3.18%)	716 (2.49%)	
Not recorded	11,462 (39.02%)	81,953 (44.92%)		11,242 (39.15%)	13,157 (45.83%)	
AST (U/L)			11.0			2.5
<40	11,740 (39.96%)	72,694 (39.85%)		11,460 (39.92%)	11,145 (38.82%)	
≥40	1010 (3.44%)	3086 (1.69%)		970 (3.38%)	1051 (3.66%)	
Not recorded	16,627 (56.60%)	106,652 (58.46%)		16,281 (56.70%)	16,515 (57.52%)	
Smoking status			10.0			16.0
Current smoker	1984 (6.75%)	10,090 (5.53%)		1962 (6.83%)	3196 (11.13%)	
Ever smoker	364 (1.24%)	2195 (1.20%)		363 (1.26%)	526 (1.83%)	
Never smoker	5432 (18.49%)	40,161 (22.01%)		5312 (18.50%)	5190 (18.08%)	
Not recorded	21,597 (73.52%)	129,986 (71.25%)		21,074 (73.40%)	19,799 (68.96%)	
Drinking status			9.0			11.6
Current drinker	335 (1.14%)	1016 (0.56%)		331 (1.15%)	416 (1.45%)	
Ever drinker	1217 (4.14%)	7739 (4.24%)		1200 (4.18%)	1786 (6.22%)	
Never drinker	6228 (21.20%)	43,691 (23.95%)		6106 (21.27%)	6710 (23.37%)	
Not recorded	21,597 (73.52%)	129,986 (71.25%)		21,074 (73.40%)	19,799 (68.96%)	
Family history of diabetes			5.0			3.7
No	28,934 (98.49%)	178,532 (97.86%)		28,271 (98.47%)	28,393 (98.89%)	
Yes	443 (1.51%)	3900 (2.14%)		440 (1.53%)	318 (1.11%)	

Values are n (%) or mean ± SD

*SD* Standardized differences, *BMI* Body mass index, *FPG* Fasting plasma glucose, *ALT* Alanine aminotransferase, *AST* Aspartate aminotransferase, *TC* Total cholesterol, *TG* Triglyceride, *HDL-C* High-density lipoprotein cholesterol, *LDL-C* Low-density lipid cholesterol, *BUN* Serum urea nitrogen, *eGFR* Estimated glomerular filtration rate

For sensitivity analyses, the inverse probability of treatment weights (IPTW) was also calculated using the estimated propensity scores. IPTW was calculated as the inverse of the propensity score for hypertensive patients and as the inverse of (1- propensity score) for the non-hypertensive patients. IPTW model was used to generate a weighted cohort [34]. We conducted a series of sensitivity analyses to evaluate the robustness of the findings of the study and how our conclusions can be affected by applying various association inference models. We added two association inference models in the original cohort and the weighted cohort in the sensitivity analysis. The calculated effect sizes and *p* values from all these models were reported and compared. All results are reported according to the STROBE statement [35, 36].

All of the analyses were performed with the statistical software package R (http://www.R-project.org, The R Foundation) and Empower-Stats (http://www.empowerstats.com, X&Y Solutions, Inc., Boston, MA). The tests were 2-tailed, and *P* < 0.05 was taken as statistically significant.

## Results

### Study population

We identified 211,809 participates (54.82% men and 45.18% women) who met our inclusion criteria (Fig. 1) of whom 29,377 (13.87%) with hypertension and 182,432 (86.13%) without hypertension. The mean age of the population was 42.10 ± 12.65 years. A total of 4173 participants developed diabetes during the median follow-up of 3.12 years. Before propensity-score matching, there were differences in several baseline characteristics between the hypertensive and non-hypertensive groups (Table 1). We found that participants with hypertension generally had higher age, BMI, FPG, TC, TG, ALT and BUN. Participants with hypertension also had a higher percentage of males and higher rates of current smokers and drinkers. With the use of one-to-one propensity-score matching, 28,711 hypertensive patients matched with 28,711 non-hypertensive subjects. After matching, the standardized differences were less than 20.0% for almost all variables, indicating that the propensity scores were well matched. Namely, there were only small differences in baseline characteristics between hypertensive and non-hypertensive groups. In addition, there were only small differences in baseline characteristics between the two groups in the weighted cohort. (Table S1).

### The incidence rate of diabetes

Table 2 showed the incidence of diabetes by hypertension exposure before and after propensity-score matching. Before propensity-score matching, a total of 4173 participants developed diabetes during follow-up. The incidence rate of diabetes was 630.947 per 100,000 person-years in the overall population, 1693.144 per 100,000 person-years in the hypertensive group and 460.303 per 100,000 person-years in the non-hypertensive group. The corresponding cumulative incidence of diabetes in the hypertension and non-hypertension groups was 5.276% (5.021–5.532%) and 1.438% (1.383–1.492%), respectively. This difference in the morbidity between the two groups changed significantly after the PS-matching procedure (1576.280 per 100,000 person-years among the overall population, 1614.820 per 100,000 person-years among the hypertensive subjects and 1538.463 per 100,000 person-years among the non-hypertensive subjects). The corresponding cumulative incidence in the hypertension and non-hypertension group was 5.033% (4.780–5.286%) and 4.887% (4.637–5.136%), respectively. Besides, we assigned participants into subgroups based on propensity score tertile. Compared with those in a low propensity score level, participants with an increased propensity score level had a higher cumulative incidence in the original cohort (p for trend<0.00001). The correlation still exists in the propensity-score matching cohort (p for trend<0.00001).

**Table 2:** Incidence rate of incident diabetes before and after propensity-score matching

Variable	Participants(n)	DM events(n)	Cumulative incidence (%) (95% CI)	Per 100,000 person-year
Before Matching				
Total	211,809	4173	1.970 (1.911–2.029)	630.947
Hypertension	29,377	1550	5.276 (5.021–5.532)	1693.144
Non-hypertension	182,432	2623	1.438 (1.383–1.492)	460.303
PS Tertile				
Low	70,603	74	0.105 (0.081–0.129)	33.556
Medium	70,603	386	0.547 (0.492–0.601)	174.786
High	70,603	3713	5.094 (5.083–5.424)	1687.506
After Matching				
Total	57,422	2848	4.960 (4.782–5.137)	1576.280
Hypertension	28,711	1445	5.033 (4.780–5.286)	1614.820
Non-hypertension	28,711	1403	4.887 (4.637–5.136)	1538.463
PS Tertile				
Low	19,141	130	0.679 (0.563–0.796)	214.725
Medium	19,140	757	3.955 (3.679–4.231)	1253.956
High	19,141	1961	10.245 (9.815–10.675)	3280.833

*CI* Confidence interval, *DM* Diabetes mellitus, *PS* Propensity score

**Fig. 2:**
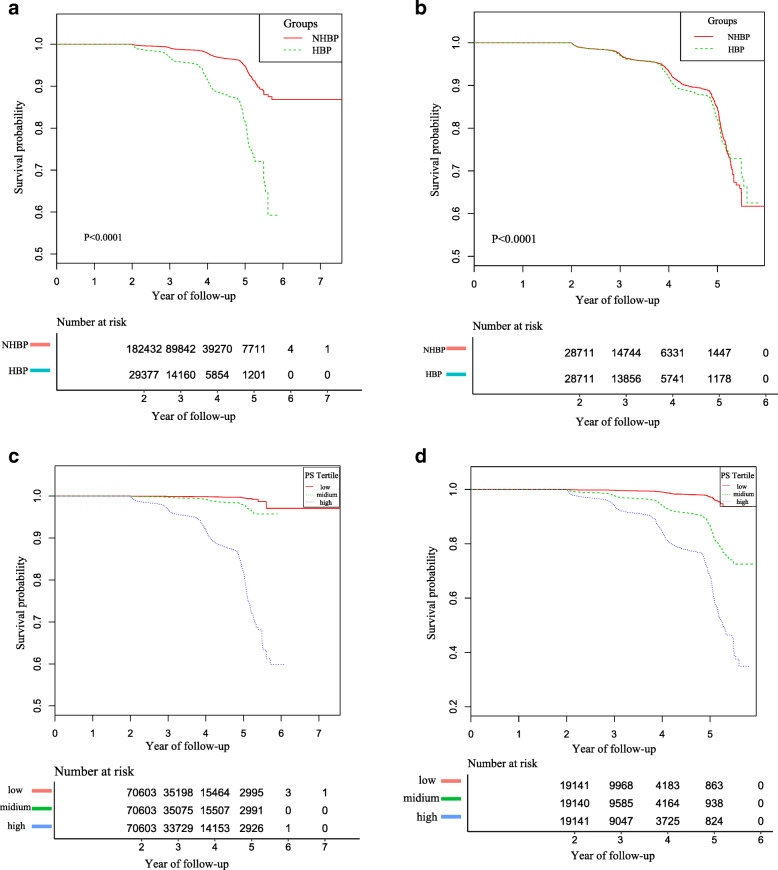
Kaplan–Meier event-free survival curve based on hypertension and propensity score tertile in the original and the propensity-score matching cohort. **a** Kaplan–Meier analysis of incident diabetes based on hypertension (HBP) and non- hypertension (NHBP) in the original cohort (log-rank, P < 0.0001).**b** Kaplan–Meier analysis of incident diabetes based on hypertension (HBP) and non- hypertension (NHBP) in the propensity-score matching cohort (log-rank, P < 0.0001). **c** Kaplan–Meier analysis of incident diabetes based on propensity score (PS) tertile in the original cohort (log-rank, *P* < 0.0001). **d** Kaplan–Meier analysis of incident diabetes based on propensity score (PS) tertile in the propensity-score matching cohort (log-rank, P < 0.0001)

Kaplan–Meier analysis demonstrated that participants with hypertension had a higher incidence of diabetes than those without hypertension in the original cohort (*P* < 0.0001). After propensity-score matching, the difference in morbidity between the two groups reduced significantly (Fig. 2).

### Association between hypertension and incident diabetes

We used the Cox proportional hazard regression model to evaluate the associations between hypertension and incident diabetes in the propensity–score–matched cohort. We simultaneously showed the results from unadjusted, minimally adjusted analysis, fully adjusted analysis and propensity-score adjusted analysis (Table 3). In crude model, hypertension had a significant correlation with incident diabetes (HR = 1.110, 95% confidence interval (CI): 1.031–1.195, *P* = 0.00539). That is, the risk of developing diabetes increased by 11.0% among hypertensive participants than those without hypertension. In the minimally adjusted model (adjusted age, gender, BMI, family history of diabetes, smoking and drinking status), the correlation still existed (HR: 1.047, 95%CI: 0.968–1.132, *P* = 0.25159). After adjusting for the full covariates (age, gender, BMI, FPG, TC, TG, HDL-C, LDL-C, ALT, AST, BUN, eGFR, family history of diabetes, smoking and drinking status), we could also detect the connection, herewith, which was not statistically significant (HR = 1.069, 95%CI: 0.988–1.157, *P* = 0.09924). In the propensity-score adjusted model, the risk of developing diabetes dropped to 8.3% in the population with hypertension (HR = 1.083, 95%CI: 1.006–1.166, *P* = 0.03367). Additionally, we explored the relation of other blood pressure indicators (SBP, DBP, pulse pressure, mean arterial pressure and hypertension grade) with incident diabetes in the original cohort, the propensity-score matching cohort and the weighted cohort. The results showed that all these blood pressure indicators are positively related to the risk of diabetes. And the risk of incident diabetes increased as the grade of hypertension increased. (Table S2).

**Table 3:** Relationship hypertension and incident diabetes in different models

Variable	Crude model (HR,95%CI,P)	Model I (HR,95%CI,P)	Model II (HR,95%CI,P)	Model III (HR,95%CI,P)
Non-hypertension	Ref.	Ref.	Ref.	Ref.
Hypertension	1.110 (1.031, 1.195) 0.00539	1.047 (0.968, 1.132) 0.25159	1.069 (0.988, 1.157) 0.09924	1.083 (1.006, 1.166) 0.03367

Crude model: we did not adjust other covariates

Model I: we adjust age, gender, BMI, family history of diabetes, smoking and drinking status

Model II: we adjust age, gender, BMI, FPG, TC, TG, HDL-C, LDL-C, ALT, AST, BUN, eGFR, family history of diabetes, smoking and drinking status

Model III: we adjust propensity score

*HR* Hazard ratios, *CI* Confidence interval, *Ref* Reference

### Subgroup analysis

**Fig. 3:**
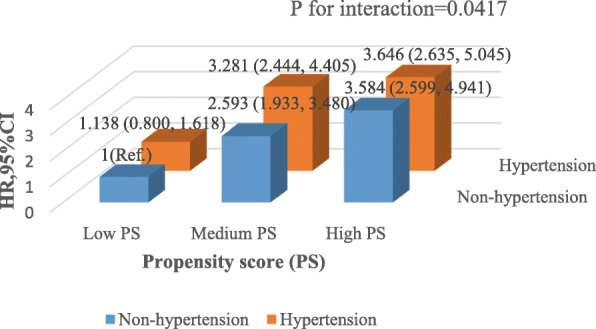
Three-dimensional bar graph for association between hypertension and non-hypertension with diabetes based on propensity score. A three-dimensional bar graph for association between hypertension and non-hypertension with diabetes based on propensity score in the propensity-score matching cohort after adjusting age, gender, BMI, FPG, TC, TG, HDL-C, LDL-C, ALT, AST, BUN, eGFR, family history of diabetes, smoking and drinking status. PS, Propensity-score; HR, Hazard ratios; CI, Confidence interval; Ref, reference

We used a subgroup analysis to detect the effect of potential confounders which may affect the relationship between hypertension and incident diabetes. We treated age, gender, BMI, FPG, eGFR, BUN, ALT, AST, TC, TG, HDL-C, LDL-C, smoking and drinking status as the stratification variables to evaluate the trend of effect sizes in these variables. Table 4 showed that none of the interactions were observed based on our prior specification. The analysis revealed that the variables listed above would not affect the association between hypertension and incident diabetes after propensity-score matching. However, we detected the interaction based on propensity score tertile (Fig. 3). Specifically, with reference to the non-hypertensive population with the low propensity score level, the hazard ratios of low, medium and high propensity score levels in the hypertensive population were 1.138 (0.800, 1.618), 3.281 (2.444, 4.405) and 3.646 (2.635, 5.045), respectively. Thus, there was a stronger association between hypertension and incident diabetes in the population with a high propensity score level.

**Table 4:** Effect size of hypertension on incident diabetes in prespecified and exploratory subgroups

Characteristic	No of participants	HR (95%CI)	P value	P for interaction
Age (years)				0.8253
< 45	10,424	1.217 (0.876, 1.691)	0.2420	
45–60	9796	1.093 (0.908, 1.315)	0.3490	
≥ 60	8134	1.047 (0.907, 1.208)	0.5317	
Gender				0.7122
Male	39,072	1.068 (0.975, 1.170)	0.1587	
Female	198	0.847 (0.341, 2.106)	0.7213	
BMI (Kg/m2)				0.2095
< 25	16,382	1.038 (0.840, 1.283)	0.7305	
≥ 25, < 29.9	12,466	1.027 (0.899, 1.175)	0.6916	
≥ 29.9	624	0.676 (0.441, 1.035)	0.0715	
FPG (mmol/L)				0.3394
< 6.1	48,902	1.162 (1.029, 1.312)	0.0154	
≥ 6.1	590	1.052 (0.774, 1.430)	0.7455	
eGFR (ml/min/1.73 m^2)				0.1310
< 90	2316	0.899 (0.678, 1.193)	0.4616	
≥ 90	42,158	1.137 (1.026, 1.260)	0.0143	
BUN (mmol/L)				0.0675
< 7.1	10,374	0.708 (0.476, 1.052)	0.0874	
≥ 7.1	26,662	1.053 (0.957, 1.159)	0.2911	
ALT(U/L)				0.7811
< 40	36,772	1.102 (0.990, 1.227)	0.0751	
≥ 40	2380	1.023 (0.743, 1.409)	0.8886	
AST(U/L)				0.5111
< 40	9038	1.123 (0.918, 1.373)	0.2607	
≥ 40	78	1.011 (0.353, 2.901)	0.9832	
Not record	18,880	0.935 (0.819, 1.066)	0.5641	
TC (mmol/L)				0.5027
< 6.22	48,008	1.092 (0.999, 1.194)	0.0521	
≥ 6.22	518	0.678 (0.312, 1.473)	0.3257	
TG (mmol/L)				0.5843
< 2.66	35,572	1.071 (0.953, 1.203)	0.2513	
≥ 2.66	3440	0.994 (0.788, 1.255)	0.9612	
HDL-C (mmol/L)				0.9791
< 1.04	588	0.984 (0.474, 2.042)	0.9659	
≥ 1.04	12,444	1.035 (0.883, 1.212)	0.6713	
Not recorded	11,022	0.953 (0.774, 1.173)	0.6496	
LDL-C (mmol/L)				0.1366
< 4.14	17,236	1.094 (0.961, 1.245)	0.1758	
≥ 4.14	54	0.000 (0.000, Inf)	0.9999	
Not recorded	10,506	1.022 (0.820, 1.274)	0.8449	
Smoking status				0.5709
Current/Ever smoker	662	1.295 (0.492, 3.412)	0.6008	
Never smoker	1954	1.134 (0.650, 1.978)	0.6581	
Not recorded	29,142	1.165 (1.055, 1.287)	0.0026	
Drinking status				0.6793
Current/Ever drinker	238	1.613 (0.446, 5.832)	0.4656	
Never drinker	2944	1.043 (0.734, 1.481)	0.8139	
Not recorded	29,142	1.165 (1.055, 1.287)	0.0026	

Note 1: Above models adjusted for age, gender, BMI, FPG, TC, TG, HDL-C, LDL-C, ALT, AST, BUN, eGFR, family history of diabetes, smoking and drinking status

Note 2: In each case, the model is not adjusted for the stratification variable

*BMI* Body mass index, *FPG* Fasting plasma glucose, *eGFR* Estimated glomerular filtration rate, *BUN* Serum urea nitrogen, *ALT* Alanine aminotransferase, *AST* Aspartate aminotransferase, *TC* Total cholesterol, *TG*, Triglyceride, *HDL-C* High-density lipoprotein cholesterol, *LDL-C* Low-density lipid cholesterol, *HR* Hazard ratios, *CI* Confidence interval

### Sensitivity analysis

We used inverse probability of treatment weights (IPTW) to generate a weighted cohort. To ensure the robustness of the results, we performed the Cox proportional hazard regression model to assess the relationship between hypertension and incident diabetes in the original cohort and the weighted cohort, respectively. Table 5 simultaneously showed the unadjusted, minimally and fully adjusted models in these two cohorts. We found that hypertension was associated with the likelihood of developing diabetes in both the original cohort and the weighted cohort. Compared with the non-hypertensive group in the full model, the risk of diabetes in the hypertensive group increased by 11.9% in the original cohort (HR = 1.119, 95%CI: 1.046–1.198, *P* = 0.00110) and 20.1% in the weighted cohort (HR = 1.201, 95%CI: 1.151–1.252, P < < 0.00001), respectively.

**Table 5:** Relationship hypertension and incident diabetes in different models of the original and the weighted cohort

Variable	Crude model (HR,95%CI, P)	Model I (HR,95%CI, P)	Model II (HR,95%CI, P)
Non-hypertension	Ref.	Ref.	Ref.
Hypertension	3.745 (3.517, 3.988) < 0.00001	1.388 (1.296, 1.486) < 0.00001	1.119 (1.046, 1.198) 0.00110
Variable	Crude model (HR,95%CI, P)	Model I (HR,95%CI, P)	Model II (HR,95%CI, P)
Non-hypertension	Ref.	Ref.	Ref.
Hypertension	1.148 (1.101, 1.197) < 0.00001	1.189 (1.140, 1.240) < 0.00001	1.201 (1.151, 1.252) < 0.00001

A In the original cohort; B In the weighted cohort

Crude model: we did not adjust other covariates

Model I: we adjust age, gender, BMI, family history of diabetes, smoking and drinking status

Model II: we adjust age, gender, BMI, FPG, TC, TG, HDL-C, LDL-C, ALT, AST, BUN, eGFR, family history of diabetes, smoking and drinking status

*HR* Hazard ratios, *CI* Confidence interval, *Ref* Reference

## Discussion

The one-to-one propensity score-matched cohort study showed that hypertension was related to a higher risk of developing diabetes in Chinese adults. After propensity-score matching, hypertension had a significant correlation with incident diabetes and the risk of developing diabetes increased by 11.0% in the population with hypertension (HR = 1.110, 95% confidence interval (CI): 1.031–1.195, *P* = 0.00539). And the figure dropped to 8.3% after adjusting the propensity score. Subgroup analysis helped us to better understand the relationship between hypertension and incident diabetes in different populations. And we found a stronger association in the population with a high propensity score level. The correlation also exists both in the original cohort and the weighted cohort.

Hypertension and diabetes share common risk factors and frequently coexist. However, there is no consensus on the association between high blood pressure and the risk of new-onset diabetes. Meanwhile, few such studies have been conducted in the Chinese population. In a study based on a cohort of 4.1 million adults published in the Journal of the American College of Cardiology, every 20 mmHg higher systolic blood pressure (SBP) was associated with a 58% higher risk of type 2 diabetes mellitus (T2DM) (HR: 1.58; 95% CI: 1.56–1.59), whereas every 10 mmHg higher diastolic blood pressure (DBP) was associated with a 52% higher risk of new-onset T2DM (HR: 1.52; 95% CI: 1.51–1.54) [37]. In the Korean genome and epidemiology study, after adjusting for some anthropometric factors, family history of diabetes and biochemical parameters, people with baseline hypertension were at higher risk of developing diabetes than the normotensive population. Specifically, in the Grade 1 hypertension group (SBP/DBP 140–159/90–99 mmHg), people had a 26% increased risk of developing diabetes (HR 1.26; 95% CI, 1.04–1.54), in the Grade 2 and 3 hypertension group (SBP/DBP ≥160/100 mmHg), people increase their risk of diabetes by 60% (HR 1.60; 95% CI,1.30–1.96) [38]. In a population-based prospective cohort study among 10,038 participants in Korea, the researchers found that compared with subjects with normal baseline blood pressure, people with baseline hypertension had a 51% higher risk of developing diabetes [39]. To the best of our knowledge, antihypertensive drugs may associate with the risk of incident diabetes [40]. However, several studies demonstrated that the increased risk of developing diabetes in people with hypertension is due to hypertension itself, given that the increased risk of diabetes persists after adjusting for specific antihypertensive treatments [6, 14]. And a study showed that for the hypertensive population under the antihypertensive treatment, SBP control in the range of 120 to < 130 mmHg, compared with the 130 to < 140 mmHg, was associated with a lower risk of incident diabetes [41]. In contrast, other studies reached inconsistent results that there was no significant association between blood pressure and the risk of incident diabetes after adjusting for some covariates [42–44]. We analyzed these inconsistent findings, and we speculated that the different results might be caused by the following factors: (1) the research population was different, including race, gender and age. (2) sample sizes in these studies varied widely. (3) these studies adjusted for different covariates which may affect the relationship between high blood pressure and diabetes risk. (4) The follow-up years varied greatly, affecting the incidence of incident diabetes. Our findings add to the existing literature, which supported the hypothesis that hypertension increased the risk of incident diabetes. Antihypertensive treatment helps control blood pressure at a relatively low level, reducing the risk of diabetes.

In the present study, the doubly robust estimation method in the propensity-score matched cohort showed a significant association between hypertension and incident diabetes. Hypertension increased the risk of developing diabetes by 11.0%. And the figure dropped to 8.3% after adjusting the propensity score. The diabetes risk in our study was relatively lower than previous researches. The difference may be that we carried out a propensity-score matching analysis that minimized potential confounders’ effect. Thus the results better showed the relationship between hypertension and diabetes in the real world. Besides, the covariates we adjusted were different. We adjusted more biochemical parameters, including FPG, TC, TG, HDL-C, LDL-C, ALT, AST, BUN and eGFR. Evidence showed that those parameters were associated with hypertension and incident diabetes [45–47]. Furthermore, our research sample is larger (211,809) and they were from 32 sites and 11 cities in China, more representative of the Chinese population. Our results supported the adverse effect of hypertension on the occurrence of diabetes. A detailed understanding of hypertension as a potential risk factor for diabetes will help us better understand and communicate risks with patients and lead to more personalized prevention and management protocols. And the propensity-score matching analysis has been mainly used to compare different treatment methods in the past [48, 49]. Our research is helpful for the promotion of propensity score methods in correlation studies.

It is still unclear whether there is a direct causal relationship between high blood pressure and diabetes risk. However, there is a substantial overlap between hypertension and diabetes in etiology and disease mechanisms. The two diseases share common mediators, including obesity, endothelial dysfunction, inflammation, oxidative stress and insulin resistance [10]. In hypertensive people, the presence of obesity leads to overactivation of the sympathetic nervous and renin-angiotensin-aldosterone systems, as well as proinflammatory/pro-oxidative mechanisms, which are related to diabetes [50, 51]. As we know, hypertension could induce endothelial dysfunction [52]. The Framingham Offspring Study revealed that some plasma markers of endothelial dysfunction (such as plasminogen activator inhibitor-1 antigen and von Willebrand factor antigen) were associated with an increased risk of new-onset diabetes independent of other diabetes risk factors including obesity, insulin resistance, and inflammation [53]. Besides, endothelial dysfunction could inhibit NO synthase and reduce NO bioavailability which is a crucial factor for the vasodilator action [54]. Therefore, endothelial dysfunction reduces vasodilation and increases vascular resistance, limiting insulin and glucose delivery in the sensitive tissues (ie, skeletal muscle, liver, and adipose tissue) and blunts insulin-stimulated glucose uptake [10, 55]. There was a low-grade inflammatory reaction in patients with diabetes and hypertension [56, 57]. High blood pressure increases inflammatory markers, such as C-reactive protein, interleukin 6 and adhesion molecules related to the insulin signaling pathway and β-cell function, and further leads to the incident diabetes [58, 59]. In addition, oxidative stress-related cytokines (interleukin-1, interleukin-6 and tumor necrosis factor-a) could modify glucose metabolism, which may contribute to the pathophysiology of diabetes mellitus [60].

Our study has some strengths. To our knowledge, so far, there are few cohort studies using propensity score matching to explore the relation of hypertension with incident diabetes. Propensity-score matching balances the distribution of measured baseline covariates to minimize measured confounding factors. Compared with other statistical methods, since the effectiveness of propensity score matching is calculated based on the average difference between matched individuals, it does not need to make any assumptions about the correlation between the dependent and explanatory variables. Meanwhile, we evaluate the relationship among comparable individuals so that our results were relatively more convincing. Furthermore, as the study was an observational study that was susceptible to the potential confusion, we also used strict statistical adjustment to further minimize residual confounders’ effect. So far, the propensity score adjustment model we conducted is rarely used. Additionally, we performed the effect modifier factor analysis to explore other potential risks of the associations between hypertension and incident diabetes in different subgroups. It is worth mentioning that we conducted a set of sensitivity analysis to ensure the reliability of the results, especially we used the inverse probability of treatment weights (IPTW) to generate a weighted cohort and further detected the association between hypertension and diabetes in the weighted cohort. Moreover, our sample size was relatively large compared to most previous similar studies, and the participants came from multiple centers.

Conversely, some limitations of our study should be noted. First of all, as the study participants were Chinese, studies of other races are needed in order to confirm that our findings can be extended to other populations. Second, we cannot obtain other important variables from the electronic database, such as the history of hypertension, antihypertensive drugs, change trajectory of blood pressure, fat distribution and weight changes (waist circumference and waist–hip ratio). And hypertension was diagnosed based on baseline blood pressure in our study. And the information about those patients with blood pressure < 140/90 under the antihypertensive treatment in primary study was not provided. Thus, the diagnostic criteria for hypertension in our study may underestimate the prevalence of hypertension. In the future, we can consider designing our studies or collaborating with other researchers to collect as many variables as possible. Third, the researchers did not conduct a 2-h oral glucose tolerance test. A study showed that just 55% of diabetic patients were diagnosed by testing fasting blood glucose alone in Asians [61]. Thus, the diagnostic criteria for diabetes in our study may underestimate the true prevalence of diabetes. However, a 2-h oral glucose tolerance test for all participants was not feasible in such a large cohort. Fourth, the raw data did not distinguish between type 1 and type 2 diabetes. However, type 2 diabetes is the most common kind of diabetes, accounting for approximately 95% of diabetes patients. Therefore, our research findings are approximately representative of type 2 diabetes [32]. And the raw data did not distinguish between primary (essential) and secondary (symptomatic) hypertension. Considering that primary (essential) hypertension is accounting for approximately 95% of patients with hypertension, so the etiological type of arterial hypertension in the study refers to primary (essential) hypertension [62]. Fifth, propensity score matching can ensure a balance of measured confounding factors but not unmeasured confounding factors. And although propensity score matching was performed based on baseline covariates to minimize measured confounders, it does not ensure that all measured baseline characteristics will match, such as gender. But we reduced the caliper width to 0.0005 to minimize the interference of some variables on the results. And more stringent caliper was also attempted but 0.0005 gave the best matching model. Sixth, this is an observational study that provides an inference of association rather than establishes a causal relationship between hypertension and diabetes. Therefore, our findings need to be interpreted cautiously and need to be further validated by prospective research.

## Conclusion

After propensity-score matching, hypertension was associated with an 11.0% increase in the risk of developing diabetes in Chinese adults. And the figure dropped to 8.3% after adjusting the propensity score. In addition, there was a stronger association in the population with a high propensity score level. Compared to non-hypertensive participants with low propensity scores, the risk of incident diabetes increased by 2.646 times among hypertensive patients with high propensity scores. Blood pressure is a potentially modifiable risk factor in terms of interventions aiming to prevent incident diabetes.

## Supplementary Information

**Additional file 1.**

## Data Availability

Data can be downloaded from ‘DATADRYAD’ database (www.Datadryad.org).

## Abbreviations

BMI: Body mass index
FPG: Fasting plasma glucose
Scr: Serum creatinine
BUN: Serum urea nitrogen
eGFR: Estimated glomerular filtration rate
TC: Total cholesterol
TG: Triglyceride
HDL-C: High-density lipoprotein cholesterol
LDL-C: Low-density lipid cholesterol
ALT: Alanine aminotransferase
AST: Aspartate aminotransferase
T2DM: Type 2 diabetes mellitus
DM: Diabetes mellitus
SD: Standardized difference
HR: hazard ratios
CI: Confidence intervals
Ref: Reference
PS: Propensity score
IPTW: Inverse probability of treatment weights
HBP: Hypertension
NHBP: Non-hypertension
SBP: Systolic blood pressure
DBP: Diastolic blood pressure
