# Supplementary material for: Association of hypertension and incident diabetes in Chinese adults: a retrospective cohort study using propensity-score matching
Source: BMC Endocr Disord. 2021 Apr 29;21:87. doi: 10.1186/s12902-021-00747-0 (PMC8082672; doi:10.1186/s12902-021-00747-0)
Supplement: Supplementary file 1 — Additional file 1. [file 12902_2021_747_MOESM1_ESM.docx]

**Table S1 Baseline characteristics in the weighted cohort.**

|  | Non-hypertension | Hypertension | SD (100%) |  |
| --- | --- | --- | --- | --- |
| Age（years） | 42.169 ± 12.631 | 42.283 ± 13.486 | 0.9 |  |
| Gender |  |  |  |  |
| Male | 55.070 | 63.070 | 16.3 |  |
| Female | 44.930 | 36.930 | 16.3 |  |
| BMI (Kg/m2) | 23.263 ± 3.379 | 23.438 ± 3.401 | 5.2 |  |
| FPG (mmol/L) | 4.918 ± 0.612 | 4.947 ± 0.633 | 4.6 |  |
| TC (mmol/L) | 4.709 ± 0.897 | 4.713 ± 0.897 | 0.4 |  |
| TG (mmol/L) | 1.343 ± 1.111 | 1.395 ± 1.001 | 4.9 |  |
| ALT (U/L) | 24.114 ± 27.388 | 25.188 ± 19.805 | 4.5 |  |
| BUN (mmol/L) | 4.661 ± 1.120 | 4.718 ± 1.190 | 4.9 |  |
| eGFR（ml/min/1.73m^2） | 109.981 ± 15.490 | 109.587 ± 16.477 | 2.5 |  |
| HDL-C(mmol/L) |  |  |  |  |
| ＜1.04 | 6.857 | 6.934 | 0.3 |  |
| ≥1.04 | 48.445 | 48.257 | 0.4 |  |
| Not recorded | 44.699 | 44.809 | 0.2 |  |
| LDL-C (mmol/L) |  |  |  |  |
| ＜4.14 | 53.993 | 53.742 | 0.5 |  |
| ≥4.14 | 1.978 | 1.892 | 0.6 |  |
| Not recorded | 44.028 | 44.365 | 0.7 |  |
| AST (U/L) |  |  |  |  |
| ＜40 | 39.924 | 41.209 | 2.6 |  |
| ≥40 | 1.931 | 2.206 | 1.9 |  |
| Not recorded | 58.146 | 56.585 | 3.2 |  |
| Smoking status |  |  |  |  |
| Current smoker | 5.699 | 6.113 | 1.8 |  |
| Ever smoker | 1.202 | 1.284 | 0.7 |  |
| Never smoker | 21.534 | 21.135 | 1.0 |  |
| Not recorded | 71.564 | 71.468 | 0.2 |  |
| Drinking status |  |  |  |  |
| Current drinker | 0.610 | 0.826 | 2.6 |  |
| Ever drinker | 4.270 | 4.370 | 0.5 |  |
| Never drinker | 23.556 | 23.337 | 0.5 |  |
| Not recorded | 71.564 | 71.468 | 0.2 |  |
| Family history of diabetes |  |  |  |  |
| No | 97.956 | 98.060 | 0.7 |  |
| Yes | 2.044 | 1.940 | 0.7 |  |

Values are n (%) or mean ± SD

SD, Standardized differences; BMI, Body mass index; FPG, Fasting plasma glucose; ALT, Alanine aminotransferase; AST, Aspartate aminotransferase; TC, Total cholesterol; TG，Triglyceride; HDL-C, High-density lipoprotein cholesterol; LDL-C, Low-density lipid cholesterol; BUN, Serum urea nitrogen; eGFR, Estimated glomerular filtration rate.

**Table S2 Relationship various blood pressure indicators and incident diabetes in different cohorts.**

**A**

| Exposure | Crude model (HR,95%CI, P) | Model I (HR,95%CI, P) | Model II (HR,95%CI, P) |
| --- | --- | --- | --- |
| SBP | 1.039 (1.038, 1.041) <0.00001 | 1.012 (1.011, 1.014) <0.00001 | 1.005 (1.003, 1.006) <0.00001 |
| DBP | 1.046 (1.044, 1.049) <0.00001 | 1.013 (1.010, 1.016) <0.00001 | 1.007 (1.004, 1.010) <0.00001 |
| PP | 1.040 (1.038, 1.042) <0.00001 | 1.011 (1.008, 1.013) <0.00001 | 1.002 (1.000, 1.004) 0.05548 |
| MAP | 1.052 (1.050, 1.055) <0.00001 | 1.016 (1.014, 1.019) <0.00001 | 1.007 (1.005, 1.010) <0.00001 |
| Hypertension grade |  |  |  |
| Non-hypertension | Ref. | Ref. | Ref. |
| Grade 1 hypertension | 3.305 (3.079, 3.549) <0.00001 | 1.336 (1.239, 1.440) <0.00001 | 1.106 (1.026, 1.192) 0.00842 |
| Grade 2 hypertension | 5.172 (4.635, 5.771) <0.00001 | 1.526 (1.361, 1.710) <0.00001 | 1.144 (1.021, 1.282) 0.02073 |
| Grade 3 hypertension | 5.832 (4.846, 7.019) <0.00001 | 1.557 (1.289, 1.880) <0.00001 | 1.185 (0.981, 1.430) 0.07777 |

**B**

| Exposure | Crude model (HR,95%CI, P) | Model I (HR,95%CI, P) | Model II (HR,95%CI, P) |
| --- | --- | --- | --- |
| SBP | 1.013 (1.011, 1.015) <0.00001 | 1.006 (1.004, 1.009) <0.00001 | 1.003 (1.001, 1.005) 0.00609 |
| DBP | 1.006 (1.002, 1.009) 0.00044 | 1.002 (0.999, 1.005) 0.15118 | 1.004 (1.000, 1.007) 0.03168 |
| PP | 1.016 (1.013, 1.018) <0.00001 | 1.008 (1.005, 1.010) <0.00001 | 1.002 (0.999, 1.005) 0.12335 |
| MAP | 1.013 (1.010, 1.016) <0.00001 | 1.006 (1.003, 1.009) 0.00021 | 1.004 (1.001, 1.007) 0.00659 |
| Hypertension grade |  |  |  |
| Non-hypertension | Ref. | Ref. | Ref. |
| Grade 1 hypertension | 0.981 (0.905, 1.064) 0.64181 | 0.987 (0.907, 1.075) 0.76294 | 1.034 (0.949, 1.128) 0.44501 |
| Grade 2 hypertension | 1.526 (1.354, 1.719) <0.00001 | 1.192 (1.054, 1.348) 0.00528 | 1.146 (1.013, 1.297) 0.02984 |
| Grade 3 hypertension | 1.766 (1.453, 2.146) <0.00001 | 1.322 (1.085, 1.611) 0.00560 | 1.190 (0.976, 1.451) 0.08616 |

**C**

| Exposure | Crude model (HR,95%CI, P) | Model I (HR,95%CI, P) | Model II (HR,95%CI, P) |
| --- | --- | --- | --- |
| SBP | 1.025 (1.024, 1.026) <0.00001 | 1.011 (1.010, 1.012) <0.00001 | 1.007 (1.006, 1.008) <0.00001 |
| DBP | 1.016 (1.015, 1.018) <0.00001 | 1.010 (1.008, 1.011) <0.00001 | 1.011 (1.009, 1.012) <0.00001 |
| PP | 1.025 (1.024, 1.026) <0.00001 | 1.010 (1.009, 1.012) <0.00001 | 1.004 (1.002, 1.005) <0.00001 |
| MAP | 1.027 (1.025, 1.028) <0.00001 | 1.013 (1.012, 1.015) <0.00001 | 1.012 (1.010, 1.013) <0.00001 |
| Hypertension grade |  |  |  |
| Non-hypertension | Ref. | Ref. | Ref. |
| Grade 1 hypertension | 0.945 (0.903, 0.990) 0.01623 | 1.079 (1.030, 1.129) 0.00130 | 1.117 (1.066, 1.170) <0.00001 |
| Grade 2 hypertension | 2.080 (1.943, 2.227) <0.00001 | 1.439 (1.344, 1.541) <0.00001 | 1.362 (1.271, 1.459) <0.00001 |
| Grade 3 hypertension | 2.905 (2.619, 3.222) <0.00001 | 1.895 (1.708, 2.103) <0.00001 | 1.707 (1.538, 1.894) <0.00001 |

A. In the original cohort; B. In the propensity-score matching cohort; C. In the weighted cohort.

SBP, Systolic blood pressure; DBP, Diastolic blood pressure; PP, Pulse pressure; MAP, Mean arterial pressure.

Crude model: we did not adjust other covariates.

Model I: we adjust age, gender, BMI, family history of diabetes, smoking and drinking status.

Model II: we adjust age, gender, BMI, FPG, TC, TG, HDL-C, LDL-C, ALT, AST, BUN, eGFR, family history of diabetes, smoking and drinking status.
